# A microwell-based impedance sensor on an insertable microneedle for real-time in vivo cytokine detection

**DOI:** 10.1038/s41378-021-00297-4

**Published:** 2021-11-26

**Authors:** Naixin Song, Pengfei Xie, Wen Shen, Hanju Oh, Yejia Zhang, Flavia Vitale, Mehdi Javanmard, Mark G. Allen

**Affiliations:** 1 Department of Electrical and Systems Engineering, University of Pennsylvania, Philadelphia, PA USA; 2 Department of Electrical and Computer Engineering, Rutgers University, Piscataway, NJ USA; 3 Department of Physical Medicine and Rehabilitation, University of Pennsylvania, Philadelphia, PA USA; 4 Department of Orthopedic Surgery, University of Pennsylvania, Philadelphia, PA USA; 5 Corporal Michael J. Crescenz Veterans Affairs Medical Center, Philadelphia, PA USA; 6 Department of Bioengineering, School of Engineering and Applied Science, University of Pennsylvania, Philadelphia, PA USA; 7 Department of Neurology, Perelman School of Medicine, University of Pennsylvania, Philadelphia, PA USA

**Keywords:** Bionanoelectronics, Sensors, Nanosensors, Biosensors, Biosensors

## Abstract

Impedance-based protein detection sensors for point-of-care diagnostics require quantitative specificity, as well as rapid or real-time operation. Furthermore, microfabrication of these sensors can lead to the formation of factors suitable for in vivo operation. Herein, we present microfabricated needle-shaped microwell impedance sensors for rapid-sample-to-answer, label-free detection of cytokines, and other biomarkers. The microneedle form factor allows sensors to be utilized in transcutaneous or transvascular sensing applications. In vitro, experimental characterization confirmed sensor specificity and sensitivity to multiple proteins of interest. Mechanical characterization demonstrated sufficient microneedle robustness for transcutaneous insertion, as well as preserved sensor function postinsertion. We further utilized these sensors to carry out real-time in vivo quantification of human interleukin 8 (hIL8) concentration levels in the blood of transgenic mice that endogenously express hIL8. To assess sensor functionality, hIL8 concentration levels in serum samples from the same mice were quantified by ELISA. Excellent agreement between real-time in vivo sensor readings in blood and subsequent ELISA serum assays was observed over multiple transgenic mice expressing hIL8 concentrations from 62 pg/mL to 539 ng/mL.

## Introduction

Cytokines are intercellular messengers that regulate important functions in the human body^1^. Monitoring cytokine profiles plays a crucial role in prediction and early disease diagnosis, as well as in fields such as cancer immunotherapy research, transplantation, autoimmunity, and infectious disease vaccine response. During immunotherapy, cytokine monitoring can provide insights into modifications in the immune system and biomarkers of early response to treatment^2,3^ and subsequent guidance for the adaptive design of clinical treatment^4^. The capability of affinity-based biosensors and related techniques for accurate specific detection of proteins makes them an attractive approach for clinical diagnoses and treatment. Among these technologies, label-based detection approaches, such as fluorescent^5^, isotopic^6^, chemiluminescent^7^, nanoparticle^8,9^, and radioactive labeling^10,11^, are widely used in protein detection due to the common availability of reagents and simple instrumentation requirements. However, these labeling strategies often alter the surface characteristics and natural activities of the query molecule, which may confound the measurement. Moreover, the labeling procedure, which is laborious and limits the number and types of query molecules that can be studied, makes its use challenging for in situ observation^12^. Therefore, highly sensitive, reliable, label-free detection techniques are of interest in the areas of protein-protein interactions, pharmaceutical analysis, and disease diagnostics^13,14^.

Key to the success of a particular biosensing technology is its selectivity, resolution, and detection limit. Dynamic range, real-time monitoring, multiplexing capability, widespread applicability, and data handling are other key determining factors^15,16^. Extensive use of cytokine sensing in healthcare will ultimately depend on the development of techniques that enable real-time in situ monitoring of cytokines with high selectivity and sensitivity. Multiple approaches to label-free affinity-based biosensors and techniques, including mass spectrometry (MS)^17^, surface plasmon resonance (SPR), and localized surface plasmon resonance (LSPR)^18,19^, microcantilevers^20–22^, quartz crystal microbalance (QCM)^23–25^, and carbon nanotubes^26,27^, have been investigated. Despite the promise of these techniques, many still require specialized instrumentation or have not been validated in real-time, in vivo applications.

Microfabrication technology has been widely utilized to create devices for the diagnosis and monitoring of diseases^28–32^. We present a microfabricated impedance-based sensing platform for label-free, in situ detection of cytokines. The sensor relies on antibody-functionalized microwells, resulting in high selectivity, a wide detection range, and picomolar sensitivity. Our previous work demonstrated impedance sensors on glass substrates, and these sensors were able to detect low concentrations of cytokines in vitro^33^. However, one limitation of these sensors is that they do not have an insertion form factor, which would be beneficial for in vivo applications. To address this limitation, the sensor has been fabricated with a microneedle shape form factor, comprising a sensor-bearing fused silica microneedle facilitating sensor delivery into desired locations in the tissue.

An insertable sensing platform should possess sufficient sensitivity and signal resolution to detect relevant changes in the parameter of interest (e.g., the concentration level of cytokine), range of detection encompassing the physiological dynamic range, and stable detection throughout insertion and submersion in bodily fluids. With this in mind, we characterized the sensitivity and selectivity of these devices in ex vivo experiments; demonstrated quantitative detection of multiple cytokines at picomolar concentrations, including measurements using target protein suspended in phosphate-buffered saline at a 1x concentration (PBS) and in undiluted serum samples; and demonstrated sensor mechanical durability allowing it to withstand intramuscular insertion deployment. We further carried out real-time, in vivo quantification of hIL8 concentration levels in the blood of transgenic mice that endogenously express hIL8.

## Results and discussion

**Fig. 1:**
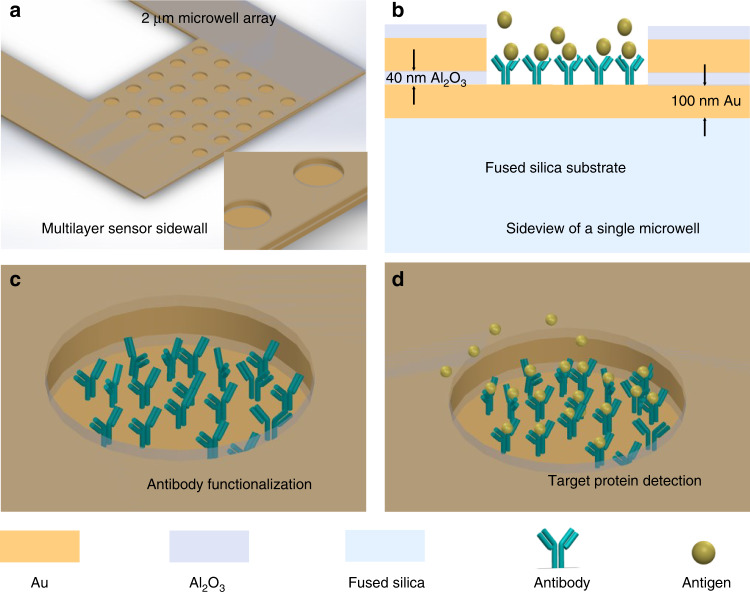
Microwell-based sensing platform. **a** A schematic view of the microwell sensor and (**b**) zoomed-in side view of the multilayer sensing structure. Schematic images showing (**c**) the antibody functionalized sensor surface inside microwell, and (**d**) specific binding of the target protein to preimmobilized antibody

The sensor, consisting of a 20 μm × 20 μm microwell array comprising 25 individual 2 μm-diameter wells embedded on a sensing tip, is lithographically configured on a laser micromachined fused silica microneedle. A schematic view of the microwell sensing platform, comprising a pair of gold electrodes separated by a 40 nm insulating layer of aluminum oxide, is shown in Fig. 1a and b. The basic principle of this impedance sensor is the continuous measurement of the impedance change between the sensor electrodes arising from the specific binding of the target protein to the probe antibody already present within the microwells. The probe antibody was first immobilized on the gold electrode surface inside the microwells, as shown in Fig. 1c. Specific binding of the target protein to this antibody (Fig. 1d) affects ion transport inside the microwells, resulting in a change in impedance between the two electrodes. As a result, real-time monitoring of both the antibody attachment process and target protein binding can be achieved by continuously capturing this change in impedance.

**Fig. 2:**
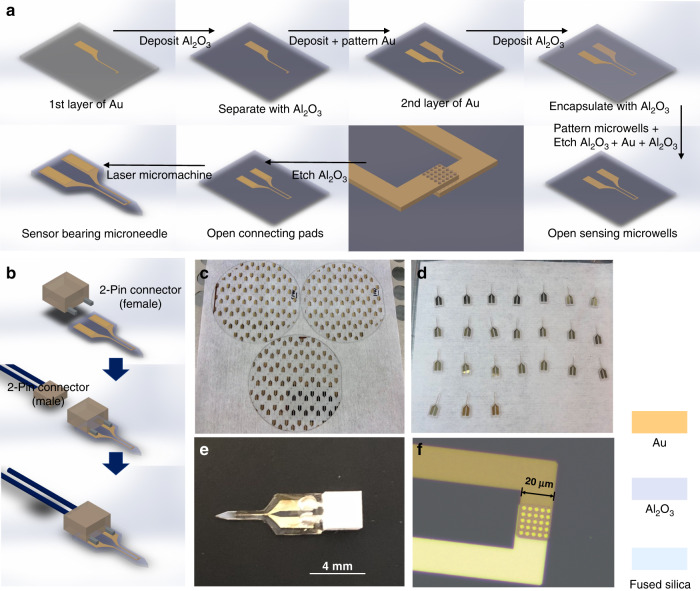
Schematic illustration and images corresponding to steps for fabricating microneedles bearing microwell sensors. **a**, **b** Steps for fabricating microwell sensors, ablating and releasing them from the substrate using an excimer laser, and packaging with connectors. Images showing **c** microscopic image of wafer-level fabricated sensors, (**d**) sensors released from the wafer after laser micromachining, (**e**) an overview of a packaged sensing platform, and (**f**) close view of tip and sensing region with an array comprising 25 individual microwells

The key sensor fabrication processes are schematically illustrated in Fig. 2a. The sensors were prepared on 100 mm diameter, 500-micron thick fused silica substrates. A first adhesion layer of 5 nm of chromium followed by 100 nm of gold was deposited by utilizing physical vapor deposition (Lesker PVD75 E-beam Evaporator) and defined by using photolithography and lift-off, yielding the lower electrode, interconnecting line, and bonding pad. The first 40 nm aluminum oxide (Al_2_O_3_) insulation layer was deposited by using atomic layer deposition (ALD, Cambridge Nanotech S200 ALD). A second 5 nm adhesion layer of chromium followed by a 100 nm gold electrode partially positioned on top of the first electrode in the sensing region, as well as a separate interconnecting line and bonding pad, was formed in the same fashion, and a second 40 nm Al_2_O_3_ layer was deposited to insulate the sensing platform. To pattern microwells (2 μm diameter) in the electrode overlapping region, a laser lithography system (Heidelberg DWL 66+ Laser Writer) was utilized. The microwells were prepared with sequential reactive ion etching (Oxford Cobra inductively coupled plasma etcher) of two layers of Al_2_O_3_ and wet etching of gold until the bottom gold electrode was exposed. Gold bonding pads were lithographically defined and exposed for connection and recording. Each sensor was micromachined into a needle shape using an excimer laser (IPG IX-255 excimer laser micromachining system) and singulated from the fused silica substrate, as shown in Fig. 2d. These microneedle sensors consist of a shank, approximately 4 mm in length and 0.9 mm in width, each bearing a two-electrode configuration. The two electrodes have an overlapping region (Fig. 2f), 20 × 20 μm in dimension, on which an array of 25 individual 2 μm diameter microwells was configured. External electrical connections to the gold bonding pads were facilitated by the use of commercially available connectors (Digi-Key Electronics, Thief River Falls, MN, USA), as shown in Fig. 2b and e. A layer of polydimethylsiloxane (PDMS, Sylgard 184, Dow Corning, 10:1 prepolymer/curing agent) was applied on the microneedle shank and connecting pads to further insulate and seal the gold interconnect traces and bonding pads.

**Fig. 3:**
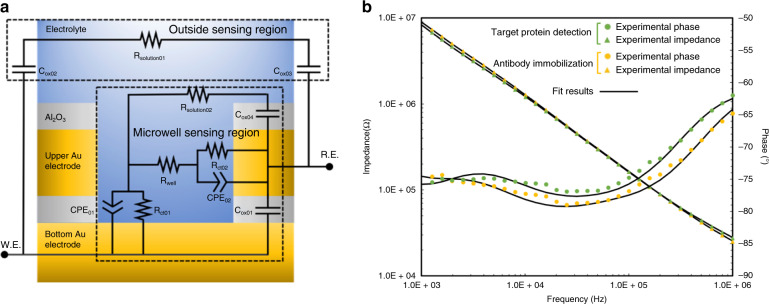
Electrochemical impedance of the microwell sensing platform. **a** A physical representation of the equivalent circuit model after the immersion of label-free sensor into electrolytes. **b** A set of representative Bode plots showing when antibody immobilization and target protein detection occurs. Experimental results are shown with symbols, and fitting results are shown with solid lines

Figure 3b shows the magnitude and phase behavior of the electrochemical impedance as a function of frequency for a typical microneedle sensor when immersed in various solution environments. In both cases, a monotonic decrease in the magnitude of impedance as a function of frequency was observed. The measured trend of the phase angle as a function of frequency was more complex. To relate these trends in electrochemical responses to the electrical properties of the microneedle sensor, the EIS data were fitted into the equivalent circuit model shown in Fig. 3a. The circuit components are hereby described with reference to the corresponding physical phenomena that they model. The equivalent circuit model is categorized into two sections indicated with dashed lines in Fig. 3a: outside sensing region and microwell sensing region. Outside sensing region: we modeled the effects of two thin oxide-insulated gold traces near the sensing region as a branch comprising a series arrangement of capacitors, C_ox02_ and C_ox03_, representing the dielectric properties of the protecting Al_2_O_3_ layers on the lower and upper gold traces, respectively, and a resistor, R_solution01_, quantifying the resistance to the movement of ions through the solution in the outside sensing region. Within the microwell sensing region, the capacitor C_ox01_ reflects the dielectric property of the Al_2_O_3_ insulation layer in the overlap region of two gold electrodes. A parallel combination of constant phase elements, C_PE01_ and C_PE02_, represents the interfacial capacitance. These interfacial capacitances, together with charge transfer resistances R_ct01_ and R_ct02_, are adopted to model the two gold–electrolyte interfaces within the microwells. R_well_ is employed to quantify the resistance to the movement of ions through the solution inside the microwells. C_ox04_ describes the dielectric property of Al_2_O_3_ on the top gold electrode, and R_solution02_ represents the resistance to the movement of ions through the solution in the region adjacent to the microwell array extending from the microwell to the uppermost Al_2_O_3_ layer.

To determine the parametric values of these circuit components, the equivalent circuit model described in Fig. 3a (solid lines in Fig. 3b) was fitted to two sets of measured representative impedance characteristics of the microneedle sensor (symbols in Fig. 3b). The fitting of measured impedance data was performed utilizing Echem Analyst, proprietary software developed by Gamry (Gamry Instruments, Warminister, PA, USA). The fitting results are represented by solid lines in Fig. 3b, and parametric values corresponding to the circuit components are summarized in Table 1. Overall, the extracted parametric values are reasonably correlated with estimated values calculated from the sensor geometry and electrical properties of materials, as listed in Table 1. By performing a perturbation analysis, we found that the goodness of fit was not highly sensitive to the parameter R_solution01_, further justifying its removal in the simplified model, as discussed in the Supplementary Information. The exploitation of the model to determine favorable impedance parameters and frequencies for use in maximizing impedance changes of the sensor with binding events is shown in the Supplementary Information. The result of this modeling showed that a suitable parameter to monitor binding event-related impedance changes of this particular sensor geometry is the real part of the sensor impedance at a frequency of 100 kHz.

**Table 1:** Summary of the EIS measurement and parameters fitted with the equivalent circuit model.

Parameter		Antibody immobilization	Target protein detection	Theoretically estimated values
R_well_ (kΩ)		47.9	63.4	/
CPE_01_ (10^−12 ^× S × s^n^)	Q_01_	75.1	84.1	55.6
	n_01_	0.869	0.847	/
R_ct01_ (MΩ)		2.12	1.97	/
CPE_02_ (10^−12 ^× S × s^n^)	Q_02_	17.3	14.3	4.5
	n_02_	0.947	0.983	/
R_ct02_ (MΩ)		75.7	73.7	/
C_ox01_ (pF)		2.46	2.46	0.7
C_ox02_ (pF)		2.95	2.95	1.8
C_ox03_ (pF)		5.14	5.14	3.5
R_solution01_ (kΩ)		29.7	36.0	/
C_ox04_ (pF)		3.83	3.83	/
R_solution02_ (kΩ)		759	819	/

**Fig. 4:**
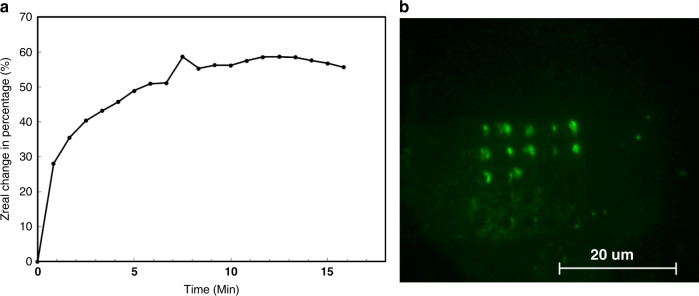
Prefunctionalizing sensors with probe antibody. **a** Real part of the sensor impedance measured at 100 kHz as a function of time during antibody adsorption. Immobilization of anti-hIL8 results in an increase in the real component of sensor impedance. **b** Optical fluorescence microscopy image of the sensor microwell region after functionalization with fluorescein-conjugated anti-hIL8 (green)

To validate the adsorption of anti-hIL8 on the sensor surface, fluorescein-conjugated anti-hIL8 (IC208G, Minneapolis, MN, USA) was employed as an adsorption probe. After incubating the sensor with this anti-hIL8 solution for 10 min, the antibody solution in the fluidic cell was removed, the cell was refilled with PBS to remove excess antibody, and the PBS rinse was removed. Consistent with the impedance monitoring result, Fig. 4b shows that anti-hIL8 antibody molecules were successfully immobilized on the sensor surface inside the microwells. In particular, antibody molecules were observed to adsorb selectively on the gold electrode surface, further illustrating that the upper Al_2_O_3_ protecting layer provided effective surface passivation against adsorption of antibody on nonmicrowell regions of the sensor. In addition, we observed that the microwell array was only partially functionalized with anti-hIL8, suggesting that the available binding sites for hIL8 might differ from sensor to sensor, which would result in a different percentage change of Z_real_ for different sensors during the antibody immobilization step. One potential explanation is that due to the nonuniformity of wafer-level fabrication, the areas of exposed gold surface inside microwells for sensors located on different regions of the substrate could differ in specific binding affinity.

**Fig. 5:**
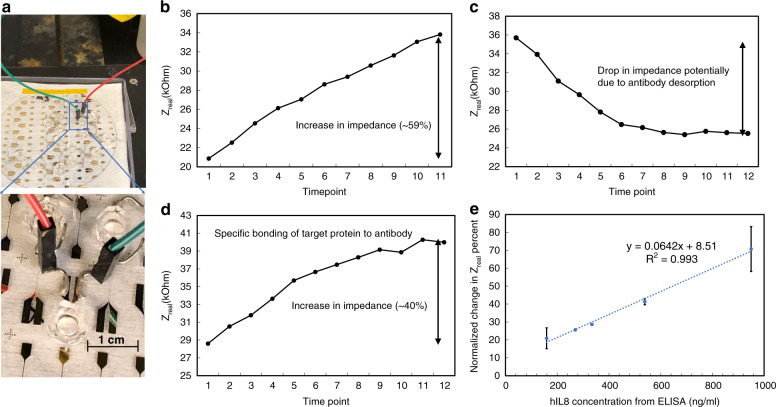
In vitro cytokine detection by microwell devices on fused silica wafers. **a** In vitro protein detection apparatus for a sensor on a fused silica wafer, comprising PDMS fluidic cell and electrical interconnection. **b**–**d** Potentiostat data at 100 kHz with a time interval of 50 s. **b** During the 10-min anti-hIL8 incubation step, the sensor responded with an increase in Z_real_. **c** After the removal of excess anti-hIL8 solution, blank PBS containing no target protein was added to the fluidic cells as a negative control, and the baseline impedance generally decreased. **d** Specific binding of target protein (hIL8) to probe the antibody (anti-hIL8) resulted in an increase in impedance after the initial baseline shift. **e** Correlation between sensor output and ELISA-based assessment of hIL8 concentration in endogenous mouse serum samples in vitro. The calibration curve showed a positive correlation between the normalized percentage change in the real component of sensor impedance $(\% \bigtriangleup {{{\mathrm{Z}}}}_{{{{\mathrm{real}}}}})$ and the hIL8 concentration. Error bars (where present) indicate the standard deviation of measurements from different sensors measured at the same concentration (*N* = 2 except at hIL8 = 1000 ng/mL, where *N* = 3)

The specific detection ability of the sensor for target antigen was investigated in vitro. Nonsingulated sensors on the fused silica substrate (Fig. 5a) were prepared with fluidic cells as described above. After the anti-hIL8 immobilization step (Fig. 5b), the fluidic cells were aspirated and rinsed using PBS. PBS containing no target protein was introduced into the cell as a negative control step. Aside from the initial baseline shift common to all steps, the impedance between the electrodes generally decreased, as shown in Fig. 5c. This was seen with all negative control experiments and was attributed to antibody desorption. Introduction of a 1 nM solution of antigen (hIL8) in PBS into the fluid cell caused the impedance to increase; the observed increase in impedance displayed an exponential time course (Fig. 5d) similar to that observed during antibody immobilization. The increase in impedance was consistent with the hypothesized reduced ion transport rates (increase in ionic resistivity) inside microwells resulting from specific bonding between the hIL8 antigen and preimmobilized anti-hIL8.

To further validate detection specificity, the microneedle sensor performance was characterized for undiluted transgenic mouse serum samples with endogenously expressed hIL8. It has been reported that mouse serum has a highly complex proteomic composition and a wide dynamic range of protein concentrations. In a previous study, more than 12,300 unique peptides that originated from 4567 unique proteins were identified^34^. These were only approximately 16% of all known mouse proteins. Quantitative measurement of the cytokine of interest against the background of these potentially confounding proteins would additionally validate the selectivity of the sensor. Samples from different mice contain different concentrations of hIL8. Sensor preparation and prefunctionalization experiments were carried out as discussed previously. In all experiments, a sample of serum from a healthy nontransgenic mouse (Sigma Aldrich, St. Louis, MO, USA) not expected to produce hIL8 was utilized as a negative control, and this was followed by the introduction of mouse serum-containing hIL8. Mouse serum samples with endogenous hIL8 generated a response similar to that of hIL8 in purified PBS buffer, as shown in Fig. 5d, while normal mouse samples exhibited behavior similar to that of PBS (as shown in Fig. 5c). Non-IL8 proteins in the mouse serum did not interfere with the ability of the sensor to quantitatively determine the presence of IL8.

Due to the nonuniform nature of wafer-level microfabrication (e.g., dry etching process to remove the Al_2_O_3_ layers and wet etching of metal thin films), sensors located on different regions of the substrate were observed to behave differently in the detection measurements. For example, sensors with differing exposed gold surface areas for immobilizing anti-hIL8 inside microwells resulted in different percentage changes in Z_real_ when these sensors interacted with solutions containing the same biological concentration level. To ameliorate this effect, the percentage change in Z_real_ during anti-hIL8 functionalization was taken to indicate the sensitivity of a particular sensor and used to calibrate subsequent responses of that particular sensor. In particular, we adopted the calibration concept of normalized percentage change in Z_real_, which is calculated as $\frac {\% \bigtriangleup {{{\mathrm{Z}}}}_{{{{{{\mathrm{real}},\,{\mathrm{detection}}}}}}}} {\% \bigtriangleup {{{\mathrm{Z}}}}_{{{{{{\mathrm{real}},\,{\mathrm{antibody}}}}}}}}$, where $\% \bigtriangleup {{{\mathrm{Z}}}}_{{\mathrm{real},\,{\mathrm{detection}}}}$ represents the percentage change in the real component of sensor impedance in the antigen detection step, and $\% \bigtriangleup {{{\mathrm{Z}}}}_{{{{{{\mathrm{real}},\,{\mathrm{antibody}}}}}}}$ represents the percentage change in the real component of sensor impedance during antibody functionalization. After measurement by the sensor, aliquots of the various mouse sera were sent for ELISA, and the correlation between the two measurement methods was assessed. Outlier testing was performed for all quantitative data. Figure 5e reveals a positive correlation between the normalized percentage change in the real component of sensor impedance and the hIL8 antigen concentration level in undiluted mouse serum. An increase in the concentration of hIL8 expedited the transfer of this antigen from the solution to the electrode surface and drove the antibody-antigen binding reaction forward. The calibration curve showed a detection range from 100 ng/mL to 1000 ng/mL, with an R^2^ of 0.993. Together, these serum sample experiments demonstrated that the sensor el ectrical measurements can be readily attributed to selective antibody-antigen binding, and they validated the ability of the microwell sensor to discriminate against false-positive signals arising from either electronic noise or nonspecific binding.

To qualitatively demonstrate the capability of microwell technology to detect other cytokine proteins with high selectivity, we functionalized the sensor with alternative antibodies, including anti-TNFα IgG (R&D Systems, Minneapolis, MN, USA) and anti-IL6 (R&D Systems, Minneapolis, MN, USA), and recorded the real component of impedance-versus-time measurements when the antibody-functionalized sensors were tested with solutions with and without the target proteins. As expected, when the target protein was present in the solution, Z_real_ increased after incubation for 10 min, whereas in the case of the negative control, the sensors responded with a decrease in Z_real_. These results illustrated that microwell technology is generalizable for monitoring other cytokines.

**Fig. 6:**
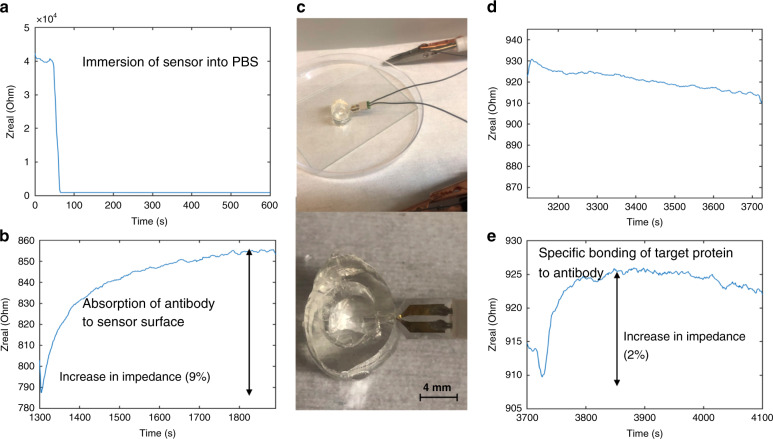
In vitro cytokine detection by a microwell sensing platform in a needle configuration. **a** Initial wetting of the sensor by adding PBS to the fluidic cell resulted in a significant drop in impedance. **b** As antibodies were physically adsorbed onto the electrode surface inside microwells, the sensor responded with an increase in impedance. **c** Microneedle was inserted through a skin phantom. **d** After sensor removal from the phantom and placement in the fluidic cell, blank PBS containing no target protein (negative control) was added to the fluidic cell, and the sensor impedance baseline generally decreased. **e** Addition of the target protein hIL8 resulted in specific binding to the probe antibody (anti-hIL8), resulting in an increase in sensor impedance after the initial baseline shift. All impedance measurements were taken at 1 MHz

The mechanical stiffness and functional stability of sensors configured on microneedles were subsequently assessed. Sensors were singulated into a microneedle shape and tested on a skin phantom to determine if the microneedle could effectively and reproducibly deliver sensors to the desired location while maintaining sensing functionality. The microneedle was first functionalized with anti-hIL8, then inserted through the skin phantom layer mimicking the dermis and hypodermis (a mixture of gelatin and agar, as shown in Fig. 6a). After insertion, the sensor was replaced back into the fluidic test cell. Figure 6b–e shows impedance data at 1 MHz obtained when the antibody-functionalized sensors were tested with PBS both with and without the target antigen.

The specific cytokine detection functionality of the sensor was further validated with a transgenic mouse model in vivo. A transgenic mouse group (positive mice) expressing hIL8 and a normal mouse group not expected to express hIL8 (negative control) were tested. Mice were anesthetized, and microneedles were inserted into the muscle without any damage, thereby successfully positioning the microwells at the desired location with an insertion depth of 3.5 mm. The sensor impedance was monitored immediately after postinsertion. Sensor-based cytokine concentration measurements were obtained using normalized percentage changes in sensor impedance, as discussed above. After sensor measurement, ELISA was performed on extracted mouse serum to quantify the concentration level of hIL8 in each mouse tested.

**Fig. 7:**
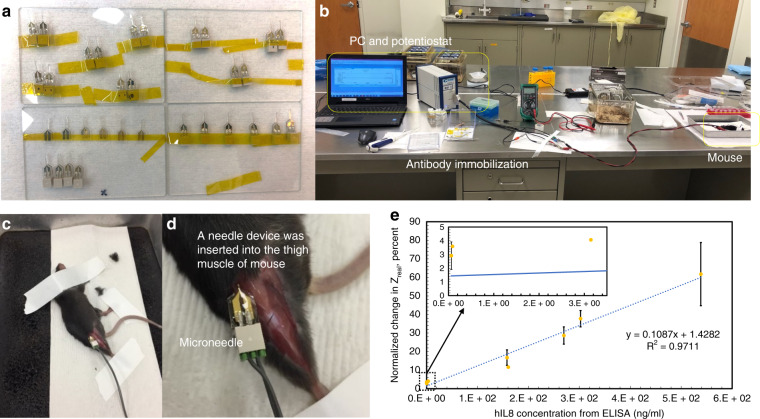
In vivo hIL8 detection by a microwell sensing platform in transgenic mice. **a** Packaged microneedle sensors ready for animal studies. **b** An in vivo experimental setup including laptops and potentiostats for data acquisition and workstations for antibody functionalization and animal operations. **c**, **d** A sensor configured on a fused silica microneedle was inserted into the thigh muscle of an anesthetized mouse. **e** Calibration curve of the linear relationship between the normalized percentage change in the real component of sensor impedance $(\% \bigtriangleup {{{\mathrm{Z}}}}_{{{{\mathrm{real}}}}})$ and hIL8 concentrations ranging from 62 pg/mL to 538.6 ng/mL. Error bars (where present) indicate the standard deviation of measurements from different sensors used at the same concentration (*N* = 2). **e** Enlarged view of the linear relationship between $\% \bigtriangleup {{{\mathrm{Z}}}}_{{{{\mathrm{real}}}}}$ and hIL8 concentration at low concentrations

Eight positive mice allowed us to determine sensor responses as a function of the concentration levels quantified by ELISA. The relationship between sensor readouts and the corresponding concentration levels is shown below in Fig. 7e. Reliable detection of hIL8 was shown for concentrations as low as one picomolar. As seen in Fig. 7e, the sensor has a wide detection range spanning four orders of magnitude, from 62 pg/mL to 538.6 ng/mL, with an R^2^ of 0.9711. Sensors in eight control mice showed behaviors similar to those for in vitro PBS control steps, i.e., no response. The comparison between these two types of mice allowed us to not only observe specific binding of the target protein in the positive mouse group but also the rejection of the effects of other endogenous mouse proteins that might be present in the blood of both groups. It is also interesting to note that the control sample of healthy mice had a full complement of endogenously expressed proteins, in addition to any extra cytokines or biomarkers that the invasion of the microneedle might cause to be released locally; however, the microwell sensor was insensitive to all of these potentially confounding factors. It was further noted that the sensitivity of the sensors in vivo experiments (as assessed by the slope of the regression line for comparison with ELISA) was larger than that of the in vitro experiments (Supplementary Information). This is potentially due to differences in operating temperatures. All in vitro characterizations were conducted at room temperature (23 °C), while the in vivo study occurred at mouse body temperature (37 °C). A higher temperature might accelerate the transport of the antigen to the sensor surface, as well as the antibody-antigen binding reaction rate, resulting in a larger amount of specific binding inside microwells to impede ion transport with the same IL8 concentration level. The detection range measured in this study was limited by the naturally expressed levels of hIL8 in this set of available transgenic mice.

An increase in IL8 expression is observed in many cancer types, such as brain, breast, cervical, colorectal^35^, gastric, lung, melanoma, ovarian, prostate, renal, oral^36^, and thyroid^37^ cancers. As a result, elevated IL8 levels have been identified as a prognostic factor^38^. In addition, it has been proven that a high concentration of IL8 is a sign of myocardial infarction^39^. Wong reported that the IL8 protein level is higher in patients with oral cancer (750 ± 236 pg/mL) than in control groups (250 ± 130 pg/mL)^40^. A serum IL8 protein value of 600 pg/mL is the threshold for discriminating patients with oral cancer from healthy individuals^41,42^. In healthy individuals, the basal IL8 clinical level is 5–10 pg/mL^43^. The present in vivo study confirmed that the detection range of the microneedle sensor for in situ hIL8 detections comfortably include the physiological dynamic range for early diagnosis of some specific types of cancer.

## Conclusion and outlook

In this study, label-free, real-time in vivo detection of cytokines was achieved by a microfabrication-enabled impedance-based sensing platform. The sensors were realized by using a combination of microfabrication for the microwells and excimer laser micromachining for the microneedle shank. These sensors rely on antibody-functionalized microwells and result in high selectivity, a wide detection range, and picomolar sensitivity. In vitro, experimental characterizations of the device demonstrated that it possessed promising selectivity, a wide dynamic sensing range, electromechanical stability, and functional robustness for potential in vivo applications. As a case study, specific detection functionality with high selectivity was first validated for sensors on wafers by utilizing both hIL8 suspended PBS buffer and undiluted mouse serum. Endogenously expressed hIL8 at varying concentrations within undiluted mouse serum was selectively detected over other native molecules present in the serum. The selective detection capability was also validated by utilizing alternate biomarkers, including IL6 and TNF-α, at a nanomolar concentration in PBS buffer.

The microwell sensing platform formed on a microneedle also addresses the issues of previous approaches, which still require specialized instrumentation or have not been shown to exhibit real-time capability. The needle shape allows the direct transdermal or intramuscular insertion, enabling real-time, in vivo measurement of cytokines directly in the blood, with no requirement for sample preparation or extraction. We demonstrated sensor functionality in mouse models that endogenously express hIL8. The quantitative nature of our measurement was confirmed with gold-standard testing by ELISA. Microneedle sensors exhibited a broad detection range from 62 pg/mL to 539 ng/mL, which exceeds the required physiological dynamic range for the early diagnosis of multiple cancers. This demonstration comprises a substantial addition to the present techniques for cytokine profile monitoring and disease diagnosis.

These favorable results are attributed to the microsized well array configuration of the sensing region and to the mechanically adaptive characteristics of the laser-micromachined fused silica microneedle. Further optimizations of needle size and microwell configuration could lead to improved performance. Furthermore, although these results have been demonstrated by using a microneedle bearing only a single sensor, the relative sizes of the microwell array and needle footprint offer the potential for multiple bioassay sensors on a single microneedle by functionalizing the surface of each sensor with distinct antibodies and forming a full sensor platform. Such multiplexed sensors could allow the assessment of more complex diseases or conditions in vivo in real-time.

The microneedle shank has dimensions of approximately 4 mm in length and 900 μm in width, and it was designed in principle to be sufficiently robust to endure transdermal insertion. There are certain approaches that could be adopted in the future to avoid damage in vivo. One is to employ a flexible substrate with a lower Young’s modulus (e.g., Parylene thin film) to reduce insertion damage and mechanical mismatch between tissue and microneedle, as we have demonstrated previously^44^. Another is to harness, e.g., silicon-based probe technologies to miniaturize the microneedle further, which could also allow for the integration of microneedles and multiplexing electronics for future investigations. However, the underlying silicon substrate may introduce parasitic issues that could demand additional attention. New sensor designs might be necessary to address this issue, such as introducing a thick oxide layer on the surface of the silicon wafer to provide further insulation, reducing the size of bonding pads, and using a silicon substrate with high resistivity (e.g., 10 kOhm*cm).

## Materials and methods

### Materials and reagents

Monoclonal human IL8/CXCL8 antibody (anti-hIL8, MAB208), recombinant human IL8/CXCL8 protein (hIL8, 208-IL), mouse TNFα antibody (MAB410), recombinant mouse TNF-α protein (410-MT), mouse IL6 antibody (MAB406), recombinant mouse IL6 protein (406 ML), and CXCL8/IL8 Alexa Fluor 488-conjugated antibody (IC208G) were purchased from R&D Systems (Minneapolis, MN, USA). A Sylgard 184 silicone elastomer kit was purchased from Dow Corning. Phosphate-buffered saline at 1× concentration (PBS, pH = 7.4) was purchased from Sigma-Aldrich (MO, USA). All other chemicals were of analytical grade.

### Skin phantom preparation for in vitro characterization

To examine the capability of these microneedle sensors to be delivered to the position of interest in tissue and validate the sensing functionality post insertion for in vivo applications, a skin phantom was fabricated. The skin phantom was constructed to mimic the dermis and hypodermis layers of human skin. A mixture of 8% gelatin (gelatin from porcine skin, G1890 powder, Sigma-Aldrich) and 1% agar (A1296 powder, Sigma-Aldrich) was utilized^45^. The gelatin and agar powders were dissolved in deionized water using separate beakers, and each beaker was heated to 100 °C. Equal amounts of liquid from each beaker were then combined and cooled to 40 °C under constant stirring. The container was then held at 4 °C until the solution formed a gel. The lateral insertion phantom was prepared by creating a disk of gelled gelatin/agar solution 11 mm in diameter and 20 mm in height; a central core 5 mm in diameter and approximately 10 mm in height was created in this disk by using a punch to partially remove the central portion of the gelatin/agar, forming a toroidal-shaped well with a wall thickness of 3 mm.

### Electrochemical characterization

EIS measurements were performed to understand the impedance behavior of sensor prefunctionalization. In addition, it is critical to capture impedance changes in real-time as biological binding events occur; thus, to further determine the optimum frequency region for performing real-time impedance monitoring with maximum sensitivity, an equivalent circuit model was proposed to analyze and predict the electrochemical responses of the sensor when interacting with different solution environments. EIS measurements were conducted using a Gamry Reference 600 potentiostat (Gamry Instruments, Warminister, PA, USA) in a two-electrode configuration at room temperature with 100 mV AC excitation. Sensors were isolated from external electromagnetic interference with a Faraday cage.

### Reagent preparation

The probe antibodies used in this study were suspended in PBS at a concentration of 0.5 mg/mL. Purified antigen samples were prepared by dissolving target antigens in PBS at concentration levels ranging from picomolar to nanomolar. In addition to the purified samples, we employed undiluted mouse serum samples obtained from transgenic mice that have endogenous target antigens to assess the ability of the sensor to detect the target protein in the presence of a potentially confounding background matrix. For comparison, the target antigen concentration level of serum samples was quantified by ELISA.

### Specific cytokine detection of sensors in vitro characterizations

The cytokine detection functionality of microwell sensors was characterized in two configurations: (a) when sensors were on the substrate and (b) post sensor release from the substrate (i.e., in microneedle form). In both situations, millimeter-sized PDMS fluidic cells were utilized to facilitate the introduction of various solutions. Prior to any protein detection or mechanical tests, the microwells were initially functionalized by exposing them to a 5 μL solution containing the desired probe antibody. The antibodies were physically adsorbed onto the sensor surface during a 10-min incubation period. The solution inside the PDMS fluidic cell was then aspirated to remove excess antibody solution. For sensors on microneedles, when the absorption process of the antibody was completed, the sensor was inserted through the skin phantom layer described above and then retrieved back to the test fluidic cell to validate the mechanical stability and functional robustness of the sensor postinsertion. Next, for both configurations, 15 μL of a solution containing no antigens (blank PBS or pure mouse serum) as a negative control, as well as an antigen solution, were sequentially applied to the fluidic cell to provide different solutions conditions. For each step, complete stabilization of the impedance across the electrodes required less than 10 min. During these events, EIS measurements were performed to monitor the change in sensor impedance (100 mV AC excitation, room temperature).

### In vivo hIL8 detection in transgenic mice by the microwell sensing platform

Transgenic mice were generated by microinjection of the transgenic construct (linearized pCALL2-hIL8 cassette) into C57BL mice. The Cre-inducible pCALL2-hIL8 transgenic mouse line was generated by our group (available from The Jackson Laboratory, JAX no. 035378)^46^. We have acquired and are currently maintaining a mouse line expressing Crerecombinase under the control of the GDF5 promoter (generously provided by Dr. David Kinsley, Stanford University)^47^. The hIL8Tg mice were bred with GDF5Cre mice to express the transgene in GDF-5-expressing tissues conditionally. hIL8 gene expression in various tissues was verified with real-time PCR. For brevity, hIL8^+^; GDF5Cre mice are shown as hIL8^+^, and hIL8^−^; GDF5Cre mice are shown as controls.

All animal experimental procedures were approved by the Institutional Animal Care and Use Committee of the Corporal Michael J. Crescenz Veterans Affairs Medical Center in Philadelphia. Mice were housed under pathogen-free conditions with environmental enrichment, with up to 5 animals per cage. Mice were housed in disposable cages (Innovive Co., San Diego, CA, USA) with Alpha Dri® bedding (Shepherd Specialty Papers, Watertown, TN, USA) under pathogen-free conditions with environmental enrichments (nestlets by Ancare, Bellmore, NY, USA) and Bio-Serv™ Mouse Igloo™ Rodent Enrichment Device (Fisher Scientific, Hampton, NH). Mice were tested for pathogens quarterly using the EAD Mouse Surveillance Plus PRIA series from Charles River Research Animal Diagnostic Services (Wilmington, MA, USA), which includes 11 common mouse viruses, 26 bacterial strains, and 7 parasitic strains. Our facility continues to be negative for all pathogens tested under the EAD Mouse Surveillance Plus PRIA testing panel. Mice were fed PicoLab diet no. 5053 (LabDiet, Fort Worth, TX, USA) provided with acidified bottled water without restriction and maintained on a 12:12-h light:dark cycle. Room temperature was kept at 70–76 °F and humidity at 30–70%.

To validate the specificity of protein detection by the microneedle sensor, two types of mice were studied: hIL8^+^; GDF5Cre mice (hIL8^+^, with elevated hIL8 in the blood) and hIL8^−^; GDF5Cre mice (controls, without detectable hIL8. These transgenic mice have different endogenous levels of hIL8 in blood, which makes it possible to test the effects of concentration on the change in impedance across the sensor electrodes. A total of 16 mice were tested with microneedle sensors: eight hIL8^+^ mice and eight control mice. All mice were sacrificed after microneedle sensor detection to obtain enough serum to quantify the concentrations of hIL8 with commercial ELISA kits, and these results were then compared with biosensor results. The surgical and measuring procedures were as follows: a single mouse was anesthetized, the skin on the thigh was precut, and the anti-hIL8 pretreatment sensor was inserted to a depth of 3.5 mm in the thigh muscle. EIS measurements were performed to conduct the real-time impedance measurements (at 100 kHz, 100 mV AC excitation). Each mouse was tested with two different microneedle sensors.

## Supplementary information

Supplemental Information_Revised

## Data Availability

The raw and processed data required to reproduce these findings are available upon request.
