# Supplementary material for: A microwell-based impedance sensor on an insertable microneedle for real-time in vivo cytokine detection
Source: Microsyst Nanoeng. 2021 Nov 26;7:96. doi: 10.1038/s41378-021-00297-4 (PMC8626445; doi:10.1038/s41378-021-00297-4)
Supplement: Supplementary file 1 — Supplemental Information_Revised [file 41378_2021_297_MOESM1_ESM.pdf]

A Microwell-Based Impedance Sensor on an Insertable Microneedle for Real-Time *In Vivo* Cytokine Detection

Naixin Song[a*], Pengfei Xie[g], Wen Shen[a], Hanju Oh[a], Yejia Zhang[c,d,f], Flavia Vitale[b,c,d,e], Mehdi Javanmard[g], Mark G. Allen[a*]

[a] Department of Electrical and Systems Engineering, [b] Department of Bioengineering, School of Engineering and Applied Science, University of Pennsylvania, Philadelphia, PA 19104, USA

[c] Department of Physical Medicine and Rehabilitation, [d] Department of Orthopaedic Surgery, [e] Department of Neurology, Perelman School of Medicine, University of Pennsylvania, Philadelphia, PA 19104, USA

[f] Corporal Michael J. Crescenz Veterans Affairs Medical Center, Philadelphia, PA 19104, USA

[g] Department of Electrical and Computer Engineering, Rutgers University, Piscataway, NJ 08854, USA

Supplementary Information

Electrochemical impedance spectroscopy characterization

A perturbation analysis of circuit model fitting

Supplementary Table 1 Summary of the EIS measurement and fitted parameters with equivalent circuit model

Parameter		Circuit model fitted values			Theoretically estimated values
		Antibody	Target protein	Target protein_ Alternative fit	
**R_{well} (kΩ)**		47.9	63.4	63.4	/
**CPE_{01} (10^{-12}×S×s^n)**	Q_{01}	75.1	84.1	84.1	55.6
	n_{01}	0.869	0.847	0.847	/
**R_{ct01} (MΩ)**		2.12	1.97	1.97	/
**CPE_{02} (10^{-12}×S×s^n)**	Q_{02}	17.3	14.3	14.3	4.5
	n_{02}	0.947	0.983	0.983	/
**R_{ct02} (MΩ)**		75.7	73.7	73.7	/
**C_{ox01} (pF)**		2.46	2.46	2.46	0.7
**C_{ox02} (pF)**		2.95	2.95	2.95	1.8
**C_{ox03} (pF)**		5.14	5.14	5.14	3.5
**$R_{solution01}$ (kΩ)**		29.7	36	32	/
**C_{ox04} (pF)**		3.83	3.83	3.83	/
**$R_{solution02}$ (kΩ)**		759	819	819	/

It is noted that the $R_{solution}$ resistors changed between the antibody immobilization and protein detection steps as listed in **Supplementary Table 1.** This change could be due to multiple factors, including small differences in the ionic strength of various solutions applied to the sensing region during different steps. However, if this occurs, one might expect $R_{solution01}$ and $R_{solution02}$ would change by the same percentage when the sensor was interacting with different solutions. In general, this was not observed. We attribute this to the observation that $R_{solution01}$ in this sensor geometry contributes negligibly to sensor impedance in comparison with C_{ox02} and C_{ox03} within the utilized EIS frequency range. As a result, the circuit model fitting is not strongly dependent on $R_{solution01}$. A perturbation analysis was performed to verify this, wherein we fixed the $R_{solution01}$ to increase by the same percentage as $R_{solution02}$ (e.g., for one data set, an increase of 8%, from 29.7 kOhm to 32 kOhm). Fitting results without and with fixing the percentage change in $R_{solution01}$ during the target protein detection step are represented by solid lines and dashed lines, respectively in **Supplementary Fig. S1** and parametric values corresponding to the circuit components are summarized in Target Protein Alternative Fit column in **Supplementary Table 1**. As expected, the goodness of fit was not highly sensitive to the parameter $R_{solution01}$, further justifying the removal of $R_{solution01}$ in the simplified circuit model.

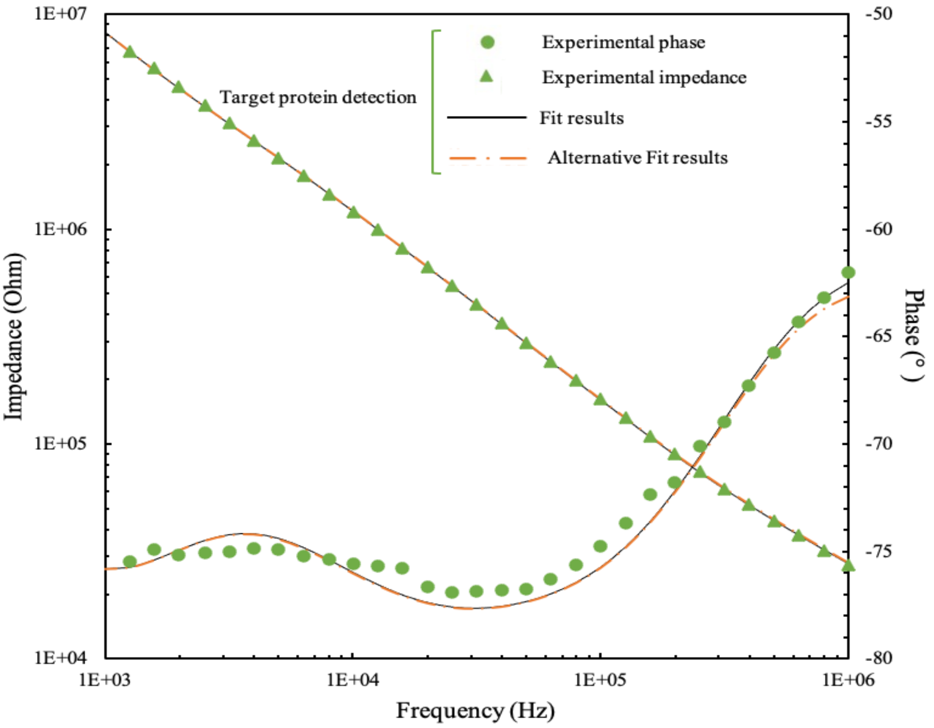

Supplementary Fig. S1 A set of representative Bode plots when target protein detection occurs. Experimental results are shown with symbols and fitting results are shown with solid lines (original fit) and dashed lines (alternative fit, in which $R_{solution01}$ is constrained).

Operating frequency determination

To illustrate the impedance behavior of sensors as biological binding events occur, and to determine a suitable operating frequency range for the sensor for real-time impedance monitoring, the equivalent circuit model was modified based on the parametric values from the above fitting results. The circuit branch comprising the outside sensing region is simplified to a single capacitor C_{ox01} as the impedance represented by $R_{solution01}$ was found to be negligible in comparison; R_{ct01}, R_{ct02} are removed as they are of many orders of magnitude larger compared to the

impedance from CPE_{01} and CPE_{02} within the range of EIS frequency as from 1kHz to 1MHz. Also, both constant phase elements have a n value that are close to 1, thus they are substituted with two double layer capacitors C_{dl01} and C_{dl02}. The simplified circuit model is shown in **Supplementary Fig. S2** The parametric values of circuit elements for this simplified model resulting from the lumped element combinations described above, as well as from a fit of the simplified model directly to the data, are summarized in **Supplementary Table 2**. Reasonable agreement between these two methods of estimation is observed, further validating the assumptions underlying the simplified model.

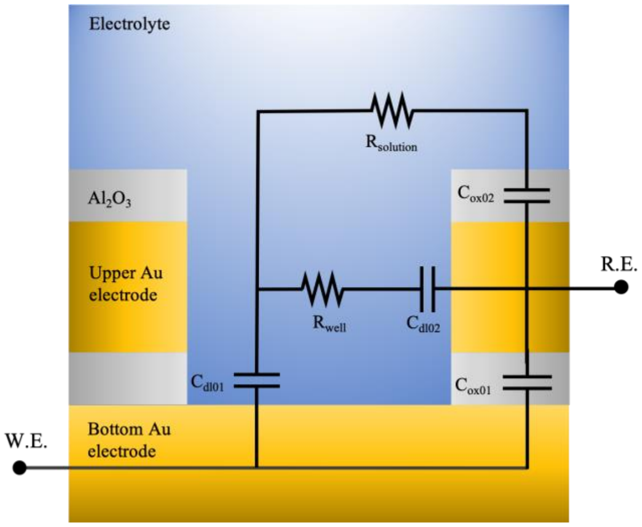

Supplementary Fig. S2 A physical representation of the simplified equivalent circuit model after the immersion of the label-free sensor into electrolytes for impedance behavior analysis.

The basic principle of this impedance sensor is that the biological binding events occurring inside microwells will influence ion transport between the two electrodes, as well as alter the dielectric properties of the electrolyte on the gold surface. As a result, the impedance behavior of the sensor will change, and real-time monitoring of the target protein can be achieved by continuously capturing this change. For example, referring to the simplified circuit model of **Supplementary Fig. S2**, it is expected that protein absorption would result in an increase in R_{well} and a decrease in C_{dl02} (as $\varepsilon_{protein}$ is ~ 20 while ε_{PBS} is ~ 80). It is further expected that these phenomena may be more or less dominant at different operating frequencies. To obtain an operating frequency region that leads to a maximum change in impedance along with the change in R_{well} and C_{dl02}, the impedance behavior of the simplified model is analyzed utilizing the parametric values of circuit components from the fitting results as summarized in **Supplementary Table 2**.

The total impedance between the gold electrodes as predicted by the simplified model of **Supplementary Fig. S2** can be expressed as:

$$Z_{total} = \frac{\left(\frac{\left(R_{well}+\frac{1}{j\omega C_{dl01}}\right)*\left(R_{solution}+\frac{1}{j\omega C_{ox02}}\right)}{R_{well}+\frac{1}{j\omega C_{dl01}}+R_{solution}+\frac{1}{j\omega C_{ox02}}}+\frac{1}{j\omega C_{dl02}}\right)*\frac{1}{j\omega C_{ox01}}}{\frac{\left(R_{well}+\frac{1}{j\omega C_{dl01}}\right)*\left(R_{solution}+\frac{1}{j\omega C_{ox02}}\right)}{R_{well}+\frac{1}{j\omega C_{dl01}}+R_{solution}+\frac{1}{j\omega C_{ox02}}}+\frac{1}{j\omega C_{dl02}}+\frac{1}{j\omega C_{ox01}}} = Z_{real} - jZ_{imag}$$

where ω is angular frequency, and Z_{real} and Z_{imag} represent the real and imaginary components of sensor impedance respectively.

Supplementary Table 2 Summary of the EIS measurement and fitted parameters with simplified equivalent circuit model

Parameter	Estimated Value	Fitted Value
**C_{ox01} (pF)**	4.18	4.18
**C_{ox02} (pF)**	6.97	6.97
**C_{dl01} (pF)**	15	6.49
**C_{dl02} (pF)**	76	55
**$R_{solution}$ (kΩ)**	800	1200
**R_{well} (kΩ)**	60	53.8

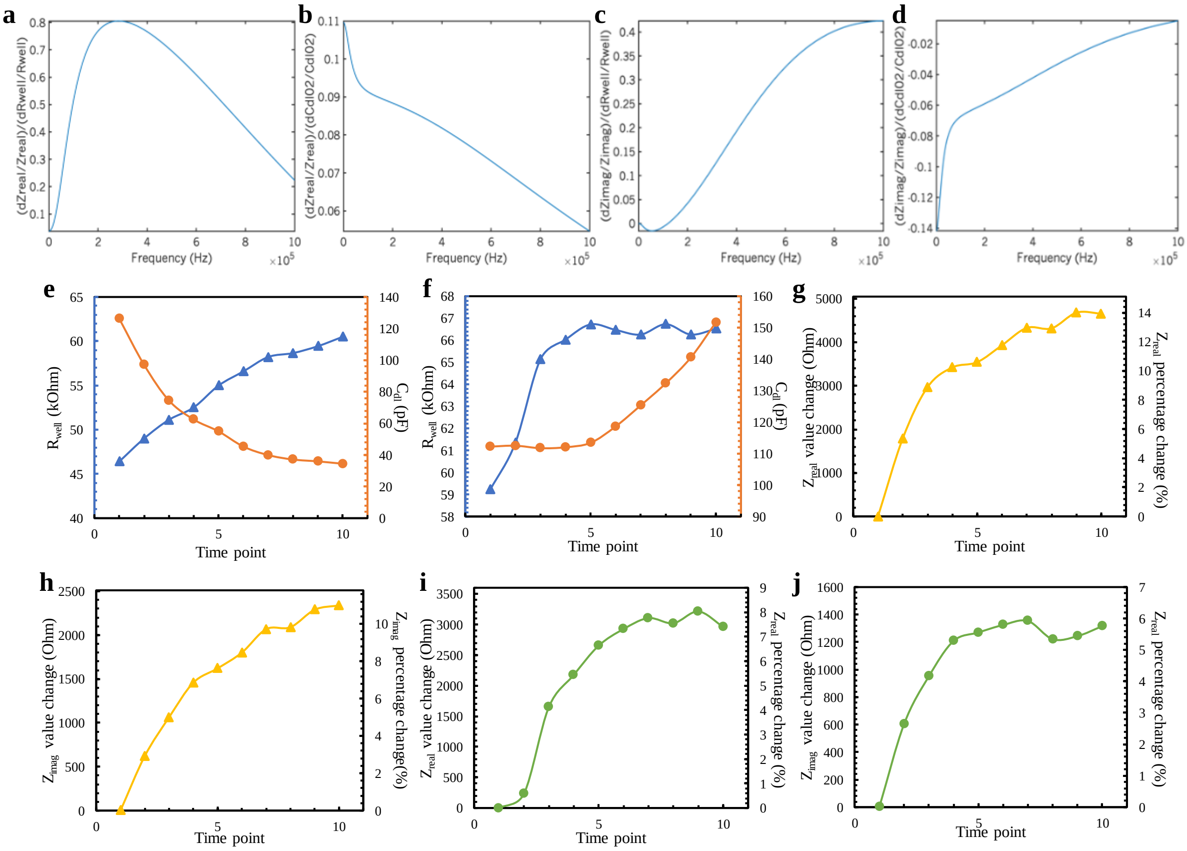

Supplementary Fig. S3 Experimental and computational analysis of impedance behavior of microwell sensing device. a-d Plots of $\frac{\partial Z_{real}/Z_{real}}{\partial R_{well}/R_{well}}$, $\frac{\partial Z_{real}/Z_{real}}{\partial C_{dlo2}/C_{dlo2}}$, $\frac{\partial Z_{imag}/Z_{imag}}{\partial R_{well}/R_{well}}$ and $\frac{\partial Z_{imag}/Z_{imag}}{\partial C_{dlo2}/C_{dlo2}}$ in terms of frequency based on the fitted parameters from equivalent circuit model. **e-f** The change of R_{well} (blue triangles) and C_{dl02} (red dots) when sensor interacted with different solutions including **e** anti-hIL8 solution and **f** hIL8 antigen suspended in PBS. **g-j** Measured changes of Z_{real} at 100kHz and Z_{imag} at 1MHz during **g, h** antibody immobilization and **i, j** target protein detection. The interval between each time point is 1 minute.

We expressed the normalized partial derivative of real and imaginary components of sensor impedance (Z_{real} and Z_{imag}) with respect to R_{well} and C_{dl02} as a function of frequency to determine an optimal operating frequency range, within which the change in R_{well} and C_{dl02} could result in the largest percentage change in either real or imaginary component of sensor impedance. The normalized partial derivative of Z_{real} and Z_{imag} with respect to R_{well} and C_{dl02} are calculated as: $\frac{\partial Z_{real}/Z_{real}}{\partial R_{well}/R_{well}}$, $\frac{\partial Z_{real}/Z_{real}}{\partial C_{dlo2}/C_{dlo2}}$, $\frac{\partial Z_{imag}/Z_{imag}}{\partial R_{well}/R_{well}}$ and $\frac{\partial Z_{imag}/Z_{imag}}{\partial C_{dlo2}/C_{dlo2}}$ using the numerical values from fitting results described in **Supplementary Table 2** and plotted in terms of frequency as shown in **Supplementary Fig.**

S3a-d. These results revealed that within a frequency range of 100 kHz -1 MHz, both Z_{real} and Z_{imag} show a relatively high sensitivity to R_{well}. **Supplementary Fig. S3a** and **c** also indicate that Z_{real} and Z_{imag} reached a peak sensitivity to R_{well} near 200 kHz and 1 MHz respectively and Z_{real} shows a higher sensitivity compared to Z_{imag} within this frequency range of interest.

To assess the ability of this simplified circuit in modeling the electrochemical properties of the sensor, a representative set of EIS data were fitted and analyzed. The EIS data were acquired when anti-hIL8 functionalization and specific binding of hIL8 to pre-immobilized anti-hIL8 were occurring within microwells. Each step included ten EIS data points within a time period of ten minutes. The changes of Z_{real} at 100 kHz and Z_{imag} at 1 MHz during antibody immobilization and target protein detection were subsequently obtained from these EIS data as shown in **Supplementary Fig. S3g-j**. The fitting routine used in this study followed a two-step procedure. First, the measured impedance data when the sensor was initially immersed in PBS were fitted to the circuit and the values of corresponding circuit components were determined (**Supplementary Table 2**). Of these, C_{ox01}, C_{ox02}, C_{dl01}, and $R_{solution}$ were assumed to be largely unaffected by biological events inside microwells. Next, the impedance data obtained during biological binding events occurring inside microwells were fitted to the model (keeping fixed the values of C_{ox01}, C_{ox02}, C_{dl01}, and $R_{solution}$) to determine the change in the values of remaining circuit components, R_{well} and C_{dl02}. **Supplementary Fig. S3e** and **f** shows the change of R_{well} and C_{dl02} when the sensor interacted with different solutions. During the anti-hIL8 functionalization step (**Supplementary Fig. S3e**), R_{well} showed an increment of 14.1 kΩ and C_{dl02} decreased from 125 pF to 34.5 pF. As discussed above, this is consistent with the proposed basic principle of the microwell sensor; i.e., the affinity of protein on the gold surface inside the microwells would block ion transport across two electrodes as well as alter the dielectric properties of the gold-electrolyte surface. When target protein solution was introduced to sensing wells, specific antibody-antigen binding occurred and an increase of 7.5 kΩ in R_{well} was observed, as shown in **Supplementary Fig. S3f**. During this step, C_{dl02} did not change as much as when anti-hIL8 was first introduced on to the gold electrode; we hypothesize that this could be due to the smaller size of the hIL8 protein molecule compared to its antibody. One potential explanation for the increase in C_{dl02} (**Supplementary Fig. 3f**) post addition of hIL8 solution might be the detachment of the anti-hIL8 from the electrode surface.

Supplementary Table 3 Summary of experimental and calculated percentage changes of Z_{real} at 100kHz and Z_{imag} at 1MHz

Percentage change		Experiment Value %	Computed Value %
**Antibody immobilization**	Z_{real} at 100kHz	13.9	8.7
	Z_{imag} at 1MHz	10.7	7.1
**Target protein detection**	Z_{real} at 100kHz	8.2	8.9
	Z_{imag} at 1MHz	5.8	3.8

To further validate the proposed circuit model, the computed percentage changes of Z_{real} and Z_{imag} based on numerical values of circuit components and change in R_{well} and C_{dl02} determined from the data fitting were compared to the measured data as summarized in **Supplementary Table 3**. Considering the fact that changing of R_{well} and C_{dl02} are in the range of 7 ~ 15 kΩ and 10 ~ 1000 pF, the influence of R_{well} dominates the change of Z_{real} and Z_{imag}. Overall, the proposed equivalent circuit models provide a good numerical fit as well as a physiological

understanding of the recorded electrochemical impedance responses of microwell sensors, which would facilitate better geometry design of sensors and optimum operating frequency region. As a result, Z_{real} within the frequency range of 100 kHz - 1 MHz was identified as optimum for real-time impedance monitoring of the microwell sensor for both *in vitro* experimental characterization and animal study in this work.

One could consider identifying the optimal operating frequency by observing the frequency with largest percentage change in impedance due to the specific binding between antigen and antibody. However, the potential for differing ionic strength of different protein solutions could be a confounding factor. To investigate further, the impedance change of one dataset between the following two time points were compared: (i) when the target antigen solution is first introduced; and (ii) and when the binding events are completed. **Supplementary Fig. S4a** and **b** present the comparisons of Z_{real} and Z_{imag} at these two time points as a function of frequency. **Supplementary Fig. S4c** shows the percentage change in Z_{real} and Z_{imag} respectively between these two time points as a function of frequency. Note that the largest changes of these parameters is observed in Z_{real} over the frequency range 50 - 500 kHz, in reasonable agreement to the frequency range derived using a circuit model combined with the EIS fitting results.

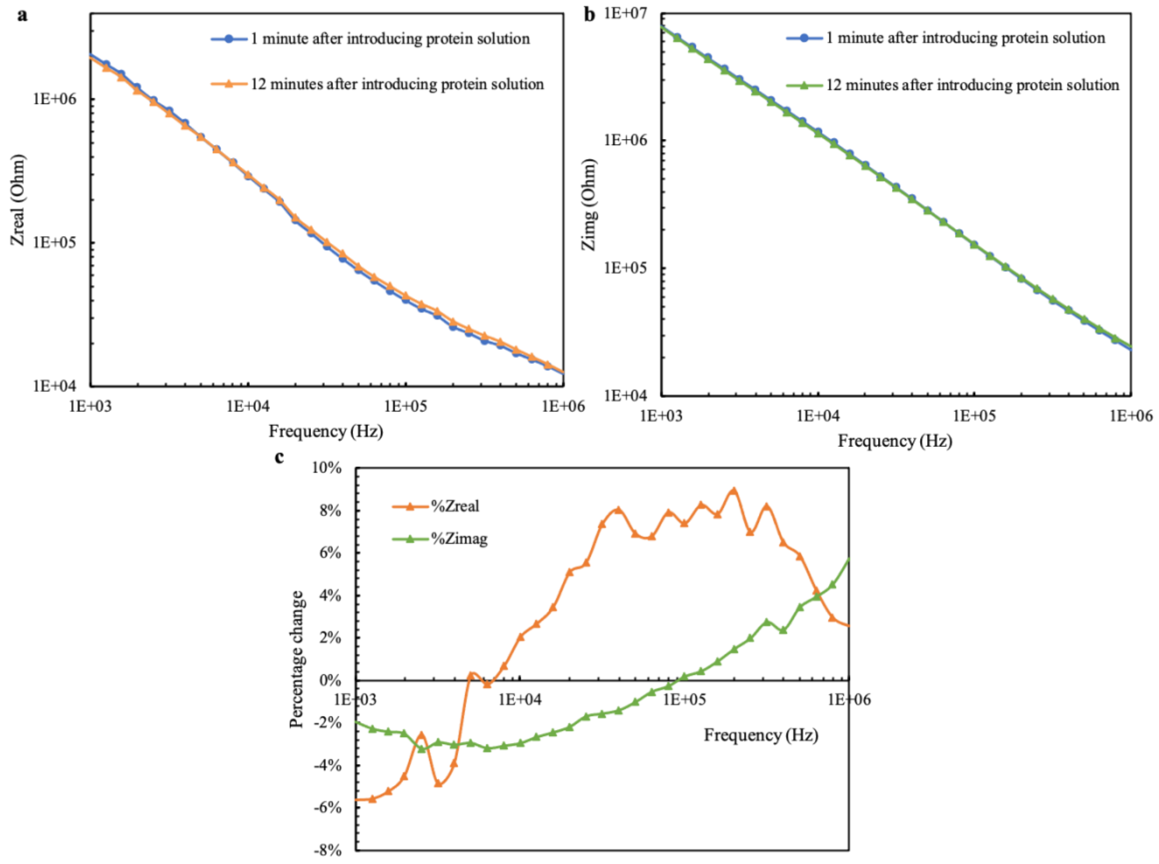

Supplementary Fig. S4 a and **b** Comparisons of **a** Z_{real} and **b** Z_{imag} at two different time points as a function of frequency; when the target antigen solution is first introduced, and when the binding events are completed. **c** Percentage change of Z_{real} and Z_{imag} between these two time points as a function of frequency.

Cytokine detection using microwell-based sensors

Specificity demonstration

In addition to mouse sera tests, *in vitro* experiments with laboratory-prepared samples, which contained a second type of cytokine protein (i.e., nontarget protein), as a negative control instead of blank PBS buffer were also conducted to assess sensor selectivity. As show in **Supplementary Fig. S5b**, the sensor was first pre-immobilized with anti-hIL8. With the introduction of nontarget protein (IL6 solution), the sensor had a negative response just as if blank PBS solution had been added. When adding the target protein solution (hIL8), the sensor responded with an increase in the Z_{real}, as expected. (We note that this particular sensor had a different electrical interconnect geometry (**Supplementary Fig. S5a**), but shared the same microwell working principle.) These results were repeatable over different protein resources, further supporting sensor specificity *in vitro.*

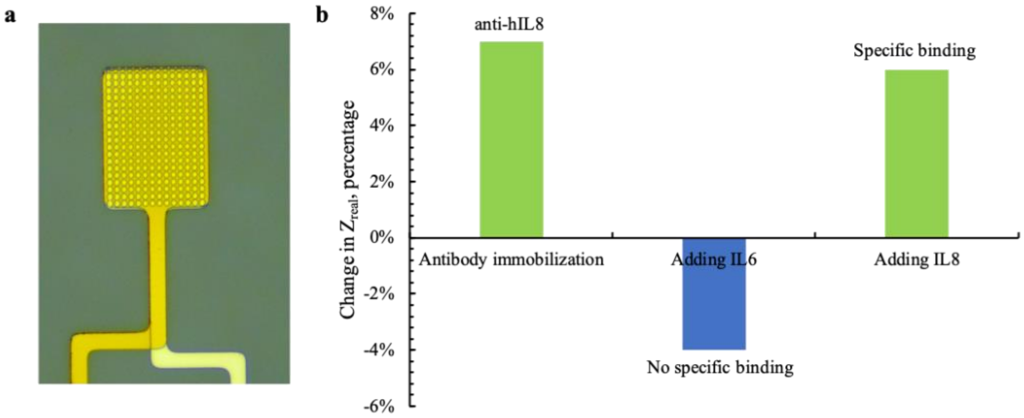

Supplementary Fig. S5 a Microscopic image of sensor sensing region with an array of microwells. **b** A summary of representative sensor responses from an experiment using IL6 solution as a negative control. Data were collected using a potentiostat at 100 kHz. Similar to blank PBS buffer, when sensor was interacting with IL6 solution, a drop in Z_{real} was observed. The sensor responded with an increment in Z_{real} following the addition of target antigen (hIL8) solution.

Effect of temperature on sensor sensitivity

Temperature plays an important role in molecular transport and reaction. Regarding temperature effects on detection results, some insight can be gained by examining the difference between the sensor sensitivity (as assessed by the slope of the regression line against ELISA assessments) in *in vitro* (room temperature, 23 ℃) and *in vivo* (mouse body temperature, 37 ℃) experiments as shown in **Supplementary Fig. S6**. It is noted that at the higher operating temperature (i.e., *in vivo*), a slightly higher sensitivity (i.e., slope of normalized percentage change in Z_{real} vs hIL8 concentration assessed by ELISA) was observed. There are several potential mechanisms to explain this sensitivity effect, such as temperature dependent biomolecule delivery rate and antigen binding reaction rate, both of which would lead to higher sensitivity at higher temperature. For *in vivo* application of the sensor, this temperature effect is somewhat ameliorated by the constant temperature environment of the body; but if the sensor is to be used in environments where the temperature is not constant, temperature compensation schemes may be considered.

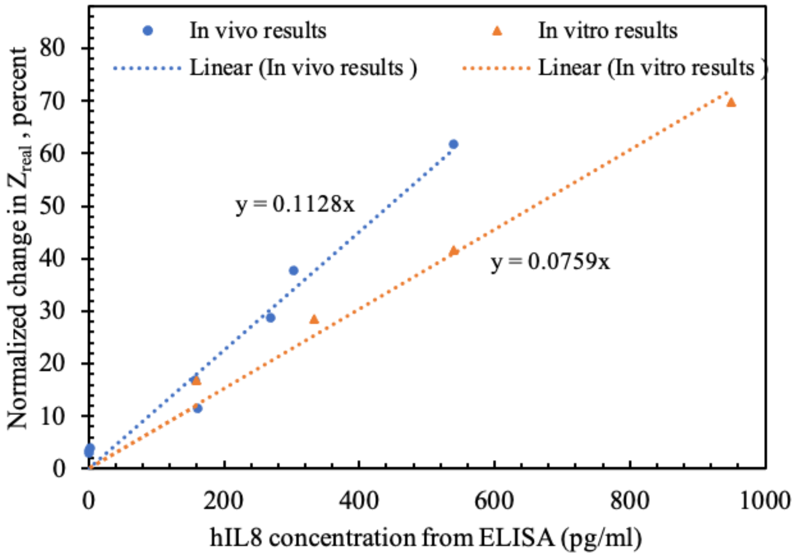

Supplementary Fig. S6 The comparison between *in vivo* (blue symbols, 37 C) and *in vitro* (red symbols, 23 C) hIL8 detection results by microwell sensing platforms.
